# Evaluation of facial cleanliness and environmental improvement activities: Lessons learned from Malawi, Tanzania, and Uganda

**DOI:** 10.1371/journal.pntd.0009962

**Published:** 2021-11-29

**Authors:** Angelia M. Sanders, Ruth Dixon, Logan Stuck, Michaela Kelly, Geordie Woods, Edridah M. Muheki, Gilbert Baayenda, Michael Masika, Holystone Kafanikhale, Upendo Mwingira, Leah Wohlgemuth

**Affiliations:** 1 The Carter Center, Atlanta, Georgia, United States of America; 2 Sightsavers, London, United Kingdom; 3 Amsterdam Institute for Global Health and Development, Amsterdam, Netherlands; 4 Uganda Ministry of Health, Kampala, Uganda; 5 Malawi Ministry of Health, Lilongwe, Malawi; 6 Tanzania Ministry of Health, Dar es Salaam, Tanzania; RTI International, UNITED REPUBLIC OF TANZANIA

## Abstract

The World Health Organization promotes the SAFE (Surgery, Antibiotics, Facial cleanliness, and Environmental improvements) strategy for trachoma control and prevention. The F&E components of the strategy focus on promotion of healthy hygiene and sanitation behaviors. In order to monitor F&E activities implemented across villages and schools in Malawi, Tanzania, and Uganda, an F&E Monitoring and Evaluation (FEME) framework was developed to track quarterly program outputs and to provide the basis for a pre and post evaluation of the activities. Results showed an increase in knowledge at the school and household levels, and in some cases, an increase in presence of hand/face washing stations. However, this did not always result in a change in trachoma prevention behaviors such as facial cleanliness or keeping compounds free of human feces. The results highlight that the F&E programs were effective in increasing awareness of trachoma prevention but not able to translate that knowledge into changes in behavior during the time between pre and post-surveys. This study also indicates the potential to improve the data collection and survey design and notes that the period of intervention was not long enough to measure significant changes.

## Author summary

Trachoma control programs promote facial cleanliness, use of latrines, and emphasizes the importance of access to water as means to reduce trachoma transmission. To address these areas, various country ministries and supporting non-governmental organizations (NGOs) support the implementation of tailored behavior change programs. The process and results of evaluating these types of programs in Malawi, Tanzania, and Uganda are described here. The goal of this manuscript is to provide trachoma program managers and supporting NGOs with insights, recommendations, and data collection tools that could be used to support their efforts to conduct monitoring and evaluation of their current or future trachoma prevention activities.

## Introduction

### Trachoma and water, sanitation, and hygiene

Trachoma, the result of ocular infection with the bacterium *Chlamydia trachomatis*, is the leading infectious cause of blindness worldwide [1]. Over time, repeated infection can cause the inside of the eyelid to become scarred and the eyelid and eyelashes to turn inward. Left untreated, the painful condition of the in-turned eyelashes scrapping the cornea can cause the individual to become irreversibly visually impaired or blind. Children have been documented to have higher levels of trachoma infection compared to other age groups, with the prevalence of active trachoma greatest among preschool age children [2–4]. The progression of the disease to the advanced blinding stage occurs over time with the onset of visual impairment typically occurring in those aged 35 years and over [5].

Transmission of the bacterium *Chlamydia trachomatis* can occur when fingers and fomites such as clothes and towels come into contact with infected ocular and nasal secretions from one individual and then contact the eyes of another individual [6]. Flies have also been documented to transmit *Chlamydia trachomatis* when the fly feeds on the ocular and nasal secretions of an infected person and then lands on the eyes of another person [7]. These same flies are strongly attracted to odors produced by human feces and lay their eggs on exposed feces on the ground [8]. Trachoma frequently affects poor and marginalized populations that typically sleep in close quarters, experience overcrowding, and lack adequate access to water and sanitation [1,9,10]. It has been challenging to determine the relative importance of one route of transmission compared to others, and it is unlikely that any single route of transmission is responsible for all trachoma transmission [6,11]. Despite not knowing the extent that Water, Sanitation, and Hygiene (WASH) programs impact trachoma prevalence within communities, certain human behaviors have been demonstrated to reduce the risk of trachoma transmission. First, reducing exposed human feces, through methods such as using pit latrines, reduces fly breeding grounds [7,8]. Second, avoiding sharing cloth with infected ocular or nasal discharge reduces the transmission of the bacterium between people [11]. Third, keeping one’s face clean from ocular and nasal secretions reduces the attractiveness of the face to the flies that feed on the secretions for nutritional purposes while simultaneously transmitting the bacterium [7]. Lastly, increasing communities’ sufficient and reliable access to water helps increase the likelihood that residents will be able to keep their faces, clothes, and bedding clean thereby reducing risk [12]. It is because of the transmission dynamics and progression of the disease that the World Health Organization (WHO) promotes the SAFE strategy (Surgery, Antibiotics, Facial cleanliness, and Environmental improvements) for trachoma control and prevention [1]. The F&E components of the strategy are focused on promoting healthy hygiene and sanitation behaviors and have a significant overlap with WASH activities. Given that the F&E components are outcomes, there are diverse types of interventions that can be used to achieve these outcomes. The lack of standardization and consistency in how these interventions are designed and implemented present a challenge in comparing across settings and clearly demonstrating effectiveness.

### Selection of F&E interventions

The trachoma control programs in Malawi, Tanzania, and Uganda received funding from The Queen Elizabeth Diamond Jubilee Trust (hereafter referred to as the Trust) for F&E activities, with additional funding provided to Tanzania from the United Kingdom’s Department For International Development (DFID) [13,14]. In order to guide decision making about how these funds should be spent the Ministry of Health in each country used a multi-step process. First, a situational analysis of WASH and trachoma programming within each country was conducted in order to understand the partners, resources, and existing WASH, trachoma, and F&E activities already taking place at the regional and district levels [15]. Second, the “All You Need for F&E” International Coalition for Trachoma Control (ICTC) toolkit was used to guide an F&E stakeholder workshop [16]. Representatives from ministries of water and sanitation, health, and education participated in this workshop alongside representatives from NGOs from the WASH, neglected tropical diseases (NTDs), and research sectors. Workshop participants identified a list of F&E activities and then participants narrowed down the number of activities based on available donor funding and what they felt should be prioritized and could be achievable in three years. Table 1 shows an overview of the different activities chosen for implementation by region within each country. A more detailed description of activities implemented within each country is provided in **S1 Text**. Ministries of health, water, and education, and supporting implementing partners implemented these interventions from July 2015 to March 2018 in Malawi, May 2016 to March 2018 in Tanzania, and May 2015 to March 2018 in Uganda. While F&E interventions targeted districts with a trachomatous inflammation-follicular (TF) prevalence over 5% (in children ages one to nine years), not all selected program activities were conducted in all villages within the targeted districts due to lack of funding and implementing partner availability.

**Table 1:** Country selected F&E activities by region.

Country	Region	Activities selected
Malawi	Central	CLTS+; SLTS+; Hand and face washing stations; and BCC at community and school level: Radio, drama, songs, and print, work with school health clubs and district level committees.
	Southern	CLTS+, SLTS+, Hand and face washing stations, BCC at community and school level: Radio, drama, songs, and print, work with school health clubs and district level committees
Tanzania	Arusha and Dodoma	School WASH through School Health Clubs; SLTS+; Social marketing; and Trachoma focused WASH education in the school curricula and mandates of teachers.
	Lindi, Manyara, Pwani	CLTS+; Social marketing to promote face washing stations; "Daily nudges” towards the adoption of healthy hygiene and sanitation practices; and Supporting National Sanitation Campaign of the Ministry of Health and Social Welfare.
Uganda	Karamoja	CLTS+; Hand and face washing stations; BCC through Mother Care Groups and school health clubs; and Adaption of school curricula to include trachoma messaging.
	Busoga	Access to water through new safe water projects (funded by alternative source), strengthening water committees; BCC: training WASH promoters at parish level to work in communities, schools, community theatre, and radio; and Adaption of school curricula to include trachoma messaging.

CLTS+ = community led total sanitation with trachoma messaging; SLTS+ = school led total sanitation with trachoma messaging; BCC = behavior change communication. Note: Community Led Total Sanitation (CLTS) is a recognized methodology for mobilizing communities to completely eliminate open defecation. Communities are facilitated to conduct their own appraisal and analysis of open defecation and define their own actions including their own building of toilets and hand and face washing stations by the communities themselves to make the community Open Defecation Free (ODF) and achieve the ‘ODF-status’. For this project, we adapted the CLTS-methodology to a school setting. School Led Sanitation (SLTS) methods encourage pupils to analyze their home situation related to WASH. The WASH situation at home is discussed with the target audience, visits made and jointly solutions are sought to improve their home situation, as part of a real-life learning cycle. Through this approach we increase the outreach of the behavior change communication from the schools into the community. As part of these projects, we made sure that the CLTS and SLTS approaches included an element on trachoma, a strong emphasis on sanitation, and a focus on hand and face washing.

### Monitoring and evaluating F&E

An F&E Monitoring and Evaluation (FEME) framework was developed for each country to assist with conducting quarterly monitoring of F&E activities and to provide the basis for a pre and post evaluation of F&E activities. The indicators used within each country’s FEME included a combination of WASH and NTD indicators identified during a Delphi consultative process [17] and country specific indicators requested by the Ministry of Health. Country specific FEMEs are provided in supplemental information (S1, S2 and S3 Tables).

The FEME can be divided into two parts: 1) the logical framework that includes outcome and output indicators; and 2) program implementation activities. Theoretically implementation of activities leads to the achievement of the desired outputs and outcomes reflected by changes in their indicators. For example, conducting community meetings about trachoma (activity) should result in an increase in the percentage of people who have knowledge of hygiene practices in relation to trachoma prevention (output) which thereby contributes to an increase in the percentage of children with clean faces (outcome). Throughout the life of the Trust funded project within the three countries the F&E activities were reported on a quarterly basis. For measuring progress on achieving outcomes and outputs, each country conducted a pre and post-survey.

### Objectives of the paper

The purpose of this paper is threefold: 1) present the methods and results of the pre and post surveys in each of the three countries; 2) discuss challenges and successes of the survey process and indicator measures selected; and 3) provide recommendations based on this experience for implementing F&E monitoring and evaluation mechanisms.

## Methods

### Ethics statement

Ethical approval was obtained for the pre and post-surveys in all three countries. In Malawi from the National Health Sciences Research Committee (Ref: #16/12/1700), in Tanzania from the National Institute for Medical Research (NIMR) National Health Ethics Review Committee (NatHREC) (Ref: NIMR/HQ/R.8a/Vol. IX/2405), and in Uganda from the Uganda National Council for Science and Technology (Ref: HS 2166). The study conducted in Uganda had additional ethical approval from Emory University (eIRB#: IRB00093647). All survey participants gave written informed consent prior to participating, head teachers provided written informed consent on behalf of school children who individually assented to take part. Assent was documented.

### Evaluation units

F&E programmatic intervention regions were used as evaluation units (EUs) with sampling of villages from a sample frame of all intervention villages. Where the scale of interventions was small (Tanzania) multiple regions were grouped into a single EU. For regions where there was both school and community programming the EUs were considered separately (Table 2).

**Table 2:** Evaluation Units (EUs).

**Community Evaluation Units**						
**Country**	**Regions within an EU**	**Number intervention villages**	**Sample target Comm/HH**	**Pre-survey 2017**	**Post-survey 2018**	**Intervention period evaluated**
Malawi	Central	390	36	January	June	16 months
Malawi	Southern	258	36			
Tanzania	Lindi, Manyara, Pwani	68	60	March	May	13 months
Uganda	Karamoja	1592	42	February	November	20 months
Uganda	Busoga	4702	40			
**School Evaluation Units**						
**Country**	**Regions within an EU**	**Number intervention schools**	**Sample target schools/ SAC**	**Pre-survey 2017**	**Post-survey 2018**	**Intervention period evaluated**
Malawi	Central	107	27	January	June	16 months
Malawi	Southern	246	28			
Tanzania	Arusha and Dodoma	20	22	March	May	13 months
Uganda	Karamoja	24	15	February	November	20 months

Comm = communities; HH = households; SAC = school age children

**Fig 1:**
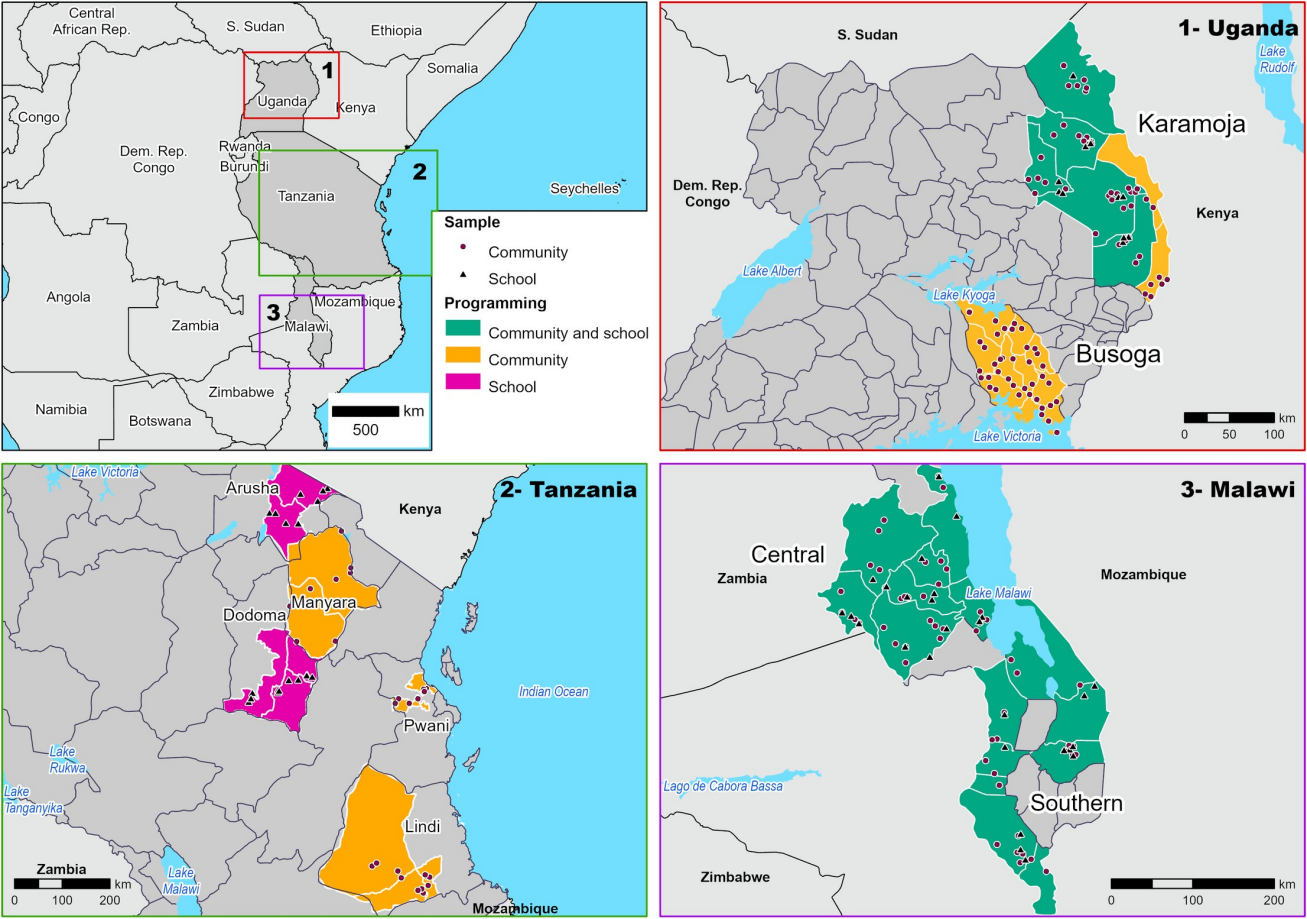
Location of F&E interventions and sampled villages and schools for pre and post-survey. Base map is from NaturalEarth for administrative boundary https://www.naturalearthdata.com/downloads/10m-cultural-vectors/ and for lake layer https://www.naturalearthdata.com/downloads/10m-physical-vectors/.

In all three countries, implementation of F&E activities began prior to the pre-surveys being conducted. Pre-surveys were conducted in 2017 and post-surveys conducted in 2018. Fig 1 provides a visual of the regions within each country where community and school activities took place and the location of the villages and schools that were selected for evaluation.

### Sampling

The sample size calculations were performed in STATA using the “sampsi” function. Sample size was based on the indicator ‘percentage of children with a clean face’ assuming 50% at baseline and the desire to detect a policy relevant change of 10 percentage points. We used 80% power in all calculations, incorporated a design effect of 2 (villages) and 1.5 (schools) and an anticipated non-response rate of 15%.

### Community sampling

Household data was collected using a population-based survey following a two-stage cluster sampling methodology. In each community EU all villages targeted for F&E interventions were listed along with their estimated population sizes. The village was the primary sampling unit and was selected using probability proportional to size. The same villages were surveyed at both pre and post-survey. The secondary sampling unit, the household, was randomly selected using a household listing approach. Households that did not have at least one adult (≥18 years in Tanzania and Uganda, ≥15 years in Malawi) who identified as the primary care giver and at least one child under nine years old were excluded from participating and replaced. Every effort was made to collect data from the selected households, which were visited three times before being replaced in cases of recurrent non-occupancy. Non-participating selected households were also replaced. Twelve villages in Lindi which had both school and community programming were excluded from the sample frames for this EU.

### School sampling

In Uganda schools were randomly selected from a list of all intervention schools in the EU. In Malawi they were selected using probability proportionate to size. In Tanzania, all intervention schools were included in the survey. In all three countries, in each selected school, two classes were randomly selected from all classes targeted with the trachoma F&E interventions. Within each of these two classes, 21 students were randomly selected for questionnaires. The same schools were used in the pre and post-surveys.

### Data collection

The surveys were designed and conducted through collaboration of partners in each country. Independent teams were recruited for the data collection who were blind to the interventions conducted and other indicators that would be generated from the data. Household questionnaires were conducted with the primary caregiver. All present household residents were directly observed for signs of ocular or nasal discharge [18]. As a proxy indicator for hand and face washing, data was collected on the presence and functionality of hand/face washing stations [19]. Direct observation of the availability of handwashing and toilet facilities was conducted. School questionnaires were conducted with head teachers along with observations of availability of toilet and handwashing facilities. Individual questionnaires were conducted with students along with observations of presence of ocular or nasal discharge on the face. Student hand and face washing behaviors were observed for a period of at least three hours in each school. In order to reduce bias we ensured that all observers/research assistants were from the respective countries, spoke the local language, and observed the events as discretely as possible. We also did not tell the schools exactly what we were observing (i.e. we did not say we were there to observe hand and face washing practices specifically). The extended observation time (3 hours) was selected in an effort to capture different opportunities in which children would wash their hands and face and also allowed the children to get used to the observer being present. There is always a risk of observer bias in the study, and we recognized this as a limitation and made as many efforts as possible to limit these biases. Household questionnaire guides were translated into the predominant local language. All questionnaires were pilot tested in the language in which it was to be conducted and revisions were made to increase clarity. School questionnaires with the head teacher were conducted in English while student ones were conducted in the local language. The English version of the questionnaires and observational data collection tools are provided in supporting information (S4, S5 and S6 Tables). Table 3 documents the different types of data collection methods used in schools and communities. With the exception of the student ‘hand/face washing behavior observation’, all observations were embedded components of the school or household questionnaire.

**Table 3:** Achieved sample size by sample characteristic at pre and post-survey.

Evaluation Unit Type	Data Collection Method	Sample Characteristic	Pre/ Post Survey	Sample Size by EU					
				Malawi		Tanzania		Uganda	
				Central	Southern	Lindi-Manyara-Pwani	Arusha-Dodoma	Karamoja	Busoga
School	School questionnaire and facility observation	Schools	Pre	28	27		21	15	
			Post	27	29		20	14	
School	Student questionnaire and face observation	Schools/ students	Pre	28/639	27/649		21/676	15/630	
			Post	27/660	29/700		20/640	14/604	
School	Hand or face washing behavior observation	Schools/ observed events	Pre	28/570	27/880		21/553	15/195	
			Post	27/1090	29/1623		20/1909	14/604	
Community	Household questionnaire and observation	Communities/ households	Pre	36/967	35/945	58/1692		43/1008	40/958
			Post	35/943	36/978	59/1696		43/1021	40/965
Community	Household face observation	Households/ faces observed	Pre	967/4493	945/4509	1692/6818		1008/5206	958/5372
			Post	943/2833	978/2817	1696/4914		1021/3052	965/3373

### Data management and analysis

Data was collected electronically using a purpose-built Open Data Kit-based Android smartphone application- LINKS for pre-survey and CommCare for post-survey [20,21]. Data was downloaded, imported to STATA13 and weighted according to sampling design and analyzed for a difference between pre and post-surveys within EUs using chi squared statistical test. A p-value of <0.05 was used to attribute a statistical difference between pre and post.

## Results of pre and post surveys

The implementation of F&E activities and quantitative evaluations of those activities were not intended as a one size fits all approach; however, common themes emerged across the three countries. In order to clearly present results of the pre and post-surveys, results are organized into the thematic groupings of WASH infrastructure, trachoma knowledge, and F&E related behaviors and are presented first for school EUs and then community EUs.

### School EU Results

Key results from each school EU within each country are provided in Table 4.

**Table 4:** Survey results by school evaluation unit at pre and post-survey.

	Malawi						Tanzania			Uganda		
	Central			Southern			Arusha and Dodoma			Karamoja		
Schools	Pre [% (95% CI)]	Post [% (95% CI)]	P	Pre [% (95% CI)]	Post [% (95% CI)]	P	Pre [% (95% CI)]	Post [% (95% CI)]	P	Pre [% (95% CI)]	Post [% (95% CI)]	P
**WASH infrastructure**												
% of schools with hand/face washing facilities with soap (1)	8.8 (2.7–25.4)	28.4 (13.8–49.6)	0.067	18.9 (4.2–55.5)	9.2 (3.0–25.0)	0.410	4.8 (0.6–30.7)	35.0 (17.3–58.0)	**0.043**	13.9 (2.9–46.8)	15.3 (3.9–44.2)	0.916
% of schools that have at least one clean latrine for both boys and girls (1)	44.8 (20.7–71.7)	70.3 (46.0–86.8)	0.157	61.0 (28.7–85.8)	78.0 (46.0–93.7)	0.395	Data not available			27.8 (9.9–57.3)	70.8 (39.1–90.2)	**0.039**
**Behavior**												
% of children with clean faces (no ocular or nasal discharge) among all children at school (2)	96.8 (93.2–98.5)	95.1 (91.5–97.2)	0.352	96.9 (92.6–98.7)	93.6 (87.5–96.9)	0.189	96.2 (88.8–98.8)	94.7 (90.6–97.1)	0.594	90.8 (81.2–95.7)	87.6 (82.2–91.5)	0.442
% of school children washing their faces when washing their hands during school day (3)	9.3 (4.8–17.4)	6.6 (2.5–16.5)	0.259	10.5 (4.2–23.6)	5.1 (1.6–14.8)	0.135	6.2 (2.3–15.9)	9.1 (5.1–15.9)	0.9	22.0 (12.2–36.2)	13.0 (6.0–25.9)	**0.048**
% of school compounds free of human feces (1)	19.6 (6.0–48.1)	92.6 (72.6–98.3)	**<0.001**	7.5 (1.9–25.0)	74.2 (28.6–95.4)	**0.004**	100.0 (100.0–100.0)	100.0 (100.0–100.0)	1.0	69.4 (37.1–89.7)	90.3 (48.1–98.9)	0.268
**Trachoma knowledge**												
% of school children who know at least one measure to prevent trachoma (2)	51.3 (44.0–58.5)	81.7 (73.5–87.8)	**<0.001**	62.8 (43.9–78.5)	66.7 (60.6–72.2)	0.67	30.2 (21.5–40.6)	49.8 (41.8–57.7)	**0.004**	49.9 (39.6–60.1)	76.3 (61.9–86.5)	**0.004**

Data collection method used: (1) school questionnaire and facility observation; (2) student questionnaire and face observation; (3) student hand/face washing behavior observation; CI = confidence interval; P = p-value

Only the EU in Tanzania showed a significant increase in the percentage of schools that had hand washing facilities with soap with 4.8% (Confidence Interval (CI): 0.6–30.7) at baseline and 35.0% (CI: 17.3–58.0) at post-survey (P = 0.043). In Uganda, there was significant increase in the percentage of schools that had at least one clean latrine for both boys and girls, with 27.8% (CI: 9.9–57.3) at baseline and 70.8% (CI: 39.1–90.2) at post-survey (P = 0.039).

There was minimal positive behavior change across the three countries. Only the two school EUs in Malawi showed a significant change in the percentage of school compounds free of human feces, with those in the Central region increasing from 19.6% (CI: 6.0–48.1) at baseline to 92.6% (72.6–98.3) at post-survey (P < 0.01) and those in the Southern region increasing from 7.5% (CI: 1.9–25.0) at baseline to 74.2% (CI: 28.6–95.4) at post-survey (P < 0.01). However, 100% of surveyed schools in Tanzania were free from human feces at baseline which was maintained through the post-survey. In Karamoja, Uganda, there was a decrease in the percentage of school children washing their faces when washing their hands during the school day with a baseline of 22.0% (CI: 12.2–36.2) decreasing to 13.0% (CI: 6.0–25.9) at post-survey (P = 0.048). There was no significant improvement in the percentage of students with a clean face between pre and post-surveys; however, the baseline percentage of children with clean face was already high across the three countries ranging from 90.8% (CI: 81.2–95.7) in Karamoja, Uganda, to 96.9% (CI: 92.6–98.7) in the Southern EU of Malawi. All but the Southern region of Malawi had a significant increase in the percentage of school children who knew at least one measure to prevent trachoma.

### Community EU Results

#### WASH Infrastructure

Key WASH infrastructure results from each EU within each country are provided in Table 5. Access to WASH infrastructure varied throughout the EUs. Only the Karamoja region of Uganda had a significant increase in the percentage of households found to have a hand/face washing station from 1.4% (CI: 0.7–2.8) at baseline to 10.1% (CI: 7.2–14.1) at post-survey (P < 0.001). In Tanzania, the percentage decreased from 13.4% (CI: 9.5–18.5) at baseline to 3.8% (CI: 2.0–7.1) at post-survey (P > 0.001). Though the Central region of Malawi did not increase the percentage of households with a hand/face washing station, they did increase the presence of soap at these washing stations. Presence of soap at the stations also increased in Karamoja and Busoga regions of Uganda, with an increase from 0.3% (CI: 0.1–0.9) at baseline to 2.3% (CI: 1.4–3.9) at post-survey (P = 0.001) in Karamoja and 2.1% (CI: 1.4–3.2) at baseline to 4.8% (CI: 2.3–9.5) at post-survey (P = 0.049) in Busoga. Only Karamoja, Uganda, had a significant change in the percentage of households with hand/face washing facilities that had water. The percentage of households with access to latrines increased in the Southern region of Malawi from 80.5% (CI: 73.3–86.1) to 88.7% (CI: 85.0–91.5) (P = 0.015) and Busoga, Uganda, from 92.3% (87.7–95.3) to 97.6% (CI: 95.6–98.7) (P > 0.003). The most dramatic increase in access to latrines was in the Karamoja region of Uganda with access increasing from 29.6% (CI: 20.2–41.0) at baseline to 93.6% (CI: 89.4–96.2) at post-survey (P < 0.001).

**Table 5:** WASH infrastructure survey results by community evaluation unit at pre and post-surveys.

	Malawi						Tanzania			Uganda					
	Central			Southern			Lindi, Manyara, Pwani			Karamoja			Busoga		
WASH infrastructure	Pre [% (95% CI)]	Post [% (95% CI)]	P	Pre [% (95% CI)]	Post [% (95% CI)]	P	Pre [% (95% CI)]	Post [% (95% CI)]	P	Pre [% (95% CI)]	Post [% (95% CI)]	P	Pre [% (95% CI)]	Post [% (95% CI)]	P
% of households found to have a hand/face washing station (1)	19.6 (13.0–28.6)	25.7 (18.6–34.3)	0.277	25.7 (15.4–39.6)	41.7 (29.6–54.8)	0.08	13.4 (9.5–18.5)	3.8 (2.0–7.1)	**0.001**	1.4 (0.7–2.8)	10.1 (7.2–14.1)	**<0.001**	11.5 (7.8–16.8)	11.8 (8.5–16.3)	0.916
% of households with hand/face washing facilities *with soap* (1)	3.7 (2.2–6.1)	10.9 (7.2–16.1)	**0.001**	7.5 (3.7–14.5)	16.3 (9.7–26.0)	0.244	2.1 (1.0–4.4)	2.1 (1.2–3.4)	0.987	0.3 (0.1–0.9)	2.3 (1.4–3.9)	**0.001**	2.1 (1.4–3.2)	4.8 (2.3–9.5)	**0.049**
% of households with hand/face washing facilities *with water* (1)	13.1 (8.2–20.2)	19.2 (13.4–26.8)	0.174	21.6 (12.2–35.3)	31.9 (20.3–46.3)	0.358	3.0 (1.8–4.9)	3.1 (1.6–5.9)	0.9	0.5 (0.2–1.2)	4.0 (2.5–6.4)	**<0.001**	5.3 (3.7–7.5)	8.4 (5.5–12.8)	0.094
% of households with access to a latrine (1)	81.4 (74.7–86.7)	81.5 (76.1–85.8)	0.986	80.5 (73.3–86.1)	88.7 (85.0–91.5)	**0.015**	78.4 (60.0–89.8)	83.4 (64.1–93.4)	0.625	29.6 (20.2–41.0)	93.6 (89.4–96.2)	**<0.001**	92.3 (87.7–95.3)	97.6 (95.6–98.7)	**0.003**

Data collection method used: (1) Household questionnaire and observation

#### Trachoma knowledge

Key results on trachoma knowledge from EUs within each country is provided in Table 6. For purposes of this manuscript, three indicators were used to measure a change in trachoma knowledge. These include: percentage of household respondents who knew one or more symptoms of trachoma; percentage of household respondents who had seen or heard any message about trachoma; and percentage of household respondents who knew one or more ways on how trachoma spreads. The Southern region of Malawi had no significant change in these three indicators. In Central Malawi, there was only an increase in percentage of respondents who knew one or more symptoms of trachoma with an increase from 39.9% (CI: 33.5–46.7) at baseline to 51.5% (CI: 42.9–60.1) at post-survey (P = 0.035). In Tanzania, there was a significant increase in percentage of household respondents who knew symptoms of trachoma and how the trachoma disease spreads. The Karamoja and Busoga regions of Uganda had a significant increase in all three indicators (P < 0.001). Additionally, it was only the Busoga region of Uganda that had a significant change in perception of personal and family risk of trachoma, which increased from 26.1% (CI: 21.3–31.6) at baseline to 38.9% (CI: 34.3–43.7) at post-survey (P > 0.001). All other evaluation units stayed approximately the same percentage between pre and post-surveys.

**Table 6:** Trachoma knowledge survey results by community evaluation unit at pre and post-surveys.

	Malawi						Tanzania			Uganda					
	Central			Southern			Lindi, Manyara, Pwani			Karamoja			Busoga		
Trachoma knowledge	Pre [% (95% CI)]	Post [% (95% CI)]	P	Pre [% (95% CI)]	Post [% (95% CI)]	P	Pre [% (95% CI)]	Post [% (95% CI)]	P	Pre [% (95% CI)]	Post [% (95% CI)]	P	Pre [% (95% CI)]	Post [% (95% CI)]	P
% of households respondents who know one or more symptoms of trachoma (1)	39.9 (33.5–46.7)	51.5 (42.9–60.1)	**0.035**	42.5 (36.3–49.1)	46.7 (40.4–53.0)	0.999	34.7 (27.3–42.9)	50.0 (44.5–55.6)	**0.003**	50.2 (44.2–56.3)	68.2 (62.7–73.3)	**<0.001**	76.9 (69.7–82.9)	97.3 (94.7–98.6)	**<0.001**
% of households respondents who have seen or heard any message about trachoma (1)	8.2 (4.5–14.5)	5.9 (4.6–7.6)	0.303	5.5 (2.9–10.2)	5.5 (2.7–11.1)	0.262	3.7 (1.8–7.6)	4.5 (2.4–8.5)	0.676	7.7 (5.5–10.6)	17.4 (13.4–22.4)	**<0.001**	6.3 (4.4–9.0)	13.7 (11.4–16.3)	**<0.001**
% of households respondents who know one or more way on how trachoma disease spreads (1)	31.8 (23.6–41.2)	39.8 (32.0–48.1)	0.184	26.0 (19.7–33.4)	32.2 (26.2–38.8)	0.187	22.8 (18.0–28.5)	35.0 (30.6–39.7)	**0.001**	25.7 (21.6–30.3)	56.1 (51.1–60.9)	**<0.001**	44.6 (38.9–50.4)	65.6 (59.9–70.8)	**<0.001**
% of respondents thinking they and family are at risk of trachoma (1)	24.7 (19.2–31.2)	25.9 (19.3–33.9)	0.798	27.7 (21.6–34.8)	23.3 (17.7–30.0)	0.323	5.3 (4.0–7.1)	6.9 (4.4–10.5)	0.321	23.0 (18.3–28.5)	24.8 (20.3–29.9)	0.615	26.1 (21.3–31.6)	38.9 (34.3–43.7)	**0.001**

Data collection method used: (1) Household questionnaire

#### F&E related behavior

Key results from EUs within each country are provided in Table 7 regarding trachoma behavior related indicators. Two indicators are reported here: clean face, defined as a face free from ocular and nasal discharge; and households free of human feces. The facial cleanliness indicator within households was broken down into three age groups: children nine years and younger; children 14 years and younger; and adults 15 years and above. This classification accommodates the nine years and younger that is typically used within trachoma programs to measure trachoma prevalence in children and the classification of adults as those 15 years and above for purposes of determining the advanced stage of trachoma (trachomatous trichiasis) in the adult population. Results across the facial cleanliness indicators for all age groups showed a significant decrease in facial cleanliness. There was no significant change in percentage of households free of human feces, with the exception of Busoga, Uganda, where there was a decrease from 96.8% (CI: 94.6–98.1) at baseline to 92.8% (CI: 90.2–94.8) at post-survey. Despite little change in this indicator across the evaluation units, it is worth noting that the percentage of households free of human feces was above 85% at baseline for most regions, ranging from 88.6% (CI: 84.0–92.1) in Karamoja, Uganda, to 96.8% (CI: 94.6–98.1) in Busoga, Uganda. In Malawi, both regions were at 95.6% at post-survey.

**Table 7:** Trachoma related behavior survey results by community evaluation unit at pre and post-survey.

	Malawi						Tanzania			Uganda					
	Central			Southern			Lindi, Manyara, Pwani			Karamoja			Busoga		
Behavior	Pre [% (95% CI)]	Post [% (95% CI)]	P	Pre [% (95% CI)]	Post [% (95% CI)]	P	Pre [% (95% CI)]	Post [% (95% CI)]	P	Pre [% (95% CI)]	Post [% (95% CI)]	P	Pre [% (95% CI)]	Post [% (95% CI)]	P
% of children ages ≤ 9 years with clean faces (no ocular or nasal discharge) among all children in or near their home (2)	76.7 (71.6–81.2)	63.9 (57.3–70.0)	**<0.001**	76.3 (71.9–80.1)	58.1 (52.5–63.6)	**<0.001**	63.4 (54.9–71.1)	62.3 (54.6–69.4)	**0.02**	60.8 (55.6–65.8)	48.7 (44.4–52.9)	**<0.001**	62.4 (57.8–66.8)	60.6 (57.0–64.0)	**0.002**
% of children ages < 15 years with clean faces (no ocular or nasal discharge) among all children in or near their home (2)	75.2 (70.3–79.5)	56.3 (50.3–62.2)	**<0.001**	73.9 (69.1–78.3)	52.0 (47.3–56.6)	**<0.001**	60.6 (53.6–67.1)	56.0 (49.6–62.2)	**0.02**	60.2 (54.8–65.3)	46.0 (42.1–49.8)	**<0.001**	58.4 (53.4–63.2)	52.0 (48.5–55.4)	**0.002**
% of adults ages ≥ 15 years with clean faces (no ocular or nasal discharge) (2)	69.9 (64.9–74.4)	56.4 (52.8–60.0)	**<0.001**	63.0 (54.2–71.0)	61.2 (57.2–65.1)	**<0.001**	67.1 (64.1–70.0)	57.8 (55.0–60.6)	**0.02**	72.0 (67.3–76.2)	56.3 (52.2–60.2)	**<0.001**	66.1 (62.2–69.7)	55.6 (52.7–58.4)	**0.002**
% of households free of human feces (1)	95.2 (92.5–97.0)	95.6 (93.5–97.0)	0.785	93.7 (89.6–96.3)	95.6 (93.7–96.9)	0.262	90.2 (81.0–95.2)	87.6 (77.7–93.5)	0.603	88.6 (84.0–92.1)	86.8 (83.1–89.7)	0.478	96.8 (94.6–98.1)	92.8 (90.2–94.8)	**0.010**

Data collection method used: (1) Household questionnaire and observation; (2) household face observation

## Discussion

In order to meet the stated objectives of the paper, the discussion is broken down into three sub-sections. First, a discussion of country specific programmatic achievements in WASH infrastructure, trachoma knowledge, and F&E related behavior. Second, an examination of the challenges and successes in the survey design and indicator measures used, and finally recommendations for future implementers.

### Programmatic achievements

#### WASH infrastructure

Data was collected on a range of WASH related indicators. For purposes of this paper, results and discussion focus on indicators that highlighted a household’s hygiene and sanitation related behaviors such as hand and face washing and latrine use, as these are believed to decrease the likelihood of trachoma transmission. As a proxy indicator for hand and face washing, data was collected on the presence and functionality of hand/face washing stations. The presence of soap and water were assumed to show an increased likelihood that the household was using the hand/face washing station. It was only in the Karamoja region of Uganda that there was a significant increase in households with hand/face washing stations and the presence of soap and water at those locations. Despite the statistically significant increases in Karamoja and a few of the other regions for select WASH indicators, programmatically the results are not encouraging. At post-survey, the overall percentages of households with hand/face washing stations ranged from a low of 3.8% in Tanzania to a high of 41.7% in the Southern region of Malawi. Hand/face washing stations with water, a likely sign of their proper use, ranged from a low of 4.0% in Karamoja to a high of 31.9% in the Southern region of Malawi. These results highlight that even where there were hand/face washing stations, the percentage that had soap and/ or water was much smaller. This could signify that simple presence of hand/face washing stations did not guarantee their use and issues revolving around access to and prioritization of water use remain.

#### Trachoma knowledge

Results showed that there was increased knowledge around trachoma in villages receiving interventions in most EUs. Though in some cases it was minimal, the results imply that at minimum F&E programs were effective at increasing knowledge within communities, a factor in behavior change programs [22]. However, it is important to recognize that knowledge is one element. The study also looked at risk perception and noted there was little change in perception of risk across the different evaluation units. It is unclear if this lack of change in perception was because the respondents felt they were putting measures in place to reduce their risk and therefore they were not concerned or if they consistently did not feel trachoma was an issue in their communities.

#### F&E related behaviors

Though the percentage of children and adults with a clean face was at least 60% across the five community-based EUs, it was not expected that the post-survey results would show a significant decrease across all age groups and regions. This could be due to a number of reasons. For example, data collection was not always standardized in the months and time of day when data was collected. The months of the pre and post-surveys varied, with some data collection occurring during the rainy season and some in the dry season when water is typically less available. This could have impacted availability of water for hand and face washing use. The time of day when clean face was documented also varied as survey teams began at one house and moved throughout the village throughout the day. Other studies have shown that time of day and physical location of data collection may impact the likelihood of a face being clean [23–25].

Ultimately the survey results from schools and households showed that though there was an increase in knowledge at the school and household level, and, in some cases, an increase in presence of hand/face washing stations, this did not always result in a measurable change in trachoma prevention behaviors such as facial cleanliness at the household level. This shows that the interventions used were effective in increasing awareness of trachoma prevention, which is a first step to changing behavior, but there remains a gap either to translate that knowledge into changes in behavior or to measure the behaviors effectively. It may also show that the data collection itself needs improvement or that the period of intervention was not long enough to measure significant changes.

## Challenges and successes

The pre and post-surveys were powered to measure changes within the EU and did not measure the success or failure of specific F&E interventions used within EUs–most of which had multiple partners and heterogenous intervention design. This means we could not compare specific F&E interventions across districts, EUs, or countries to determine what did or did not work. It is also unknown if the F&E activities were truly implemented as intended, as the only point of reference were quarterly reports submitted by F&E implementing partners to the donors. Additionally, the surveys did not include trachoma infection data, therefore, these surveys cannot claim that particular F&E related activities directly led to a decrease in trachoma prevalence.

Throughout the period of implementation of F&E activities there was not a consensus on the percentage change needed within each indicator to determine if the programs were achieving success. For example, in Tanzania, the percentage of household respondents who knew one or more ways that trachoma spreads increased statistically significantly from 22.8% to 35.0%; however, programmatically 35% would be considered a sub-optimal achievement following almost two years of F&E related programming. On the other end of the spectrum, there were instances when the baseline was already high, such as facial cleanliness in schools or household levels of sanitation. Programmatically having over 90% of school children with a clean face, greater than 78% of households having access to latrines, and 88% of households free of human feces would be considered a high level of sanitation coverage in trachoma endemic regions. For indicators that began with a high baseline, it was difficult to power statistical evidence of increase [26]. The challenge therefore is determining what the minimal thresholds are for WASH related indicators rather than purely focusing on percentage change or statistically significant changes during evaluations.

There were multiple school related indicators in the FEME. These included children having a clean face; students having access to hand/face washing stations with water and soap present; schools having functional clean latrines for staff and students; improved water sources located on premises and accessible to all users during school hours; and an awareness about how to prevent and treat trachoma. These indicators are still viewed by the authors as adequate indicators to measure F&E school-based programming for trachoma prevention and education.

There were challenges implementing the pre and post-surveys within the schools. While observations were attempted in every school, observations could not be made in schools that had no water supply or no hand/face washing facilities. In this situation, observations were made for an hour and if no hand or face washing occurred the observer proceeded to help with the school questionnaires. There was no provision in the pre-survey data collection form for recording the absence of handwashing when a student used the latrine and did not wash their hands afterwards. The student was only recorded when they did wash their hands. Therefore, a comparison between handwashing and not handwashing could not be calculated. In the post-survey, the observation form was updated and allowed for individual observations to be collected in addition to collecting when a hand/face washing event should have occurred but did not (i.e. going to the toilet but not washing hands).

In enumeration of school WASH facilities, latrines/toilets were not assessed individually for each characteristic, rather, there was a count of all latrines meeting each characteristic. This means for percentage of schools with clean and functional latrines we could not look at both clean and functional latrines together and therefore could only pick one indicator. We chose to report on cleanliness of latrines only. This decision was made due to the survey design. There were many elements that made up a functional latrine and because we did not assess each latrine individually, we could not say, for example, if the one latrine counted for having a super structure was also the one latrine counted for having a drop cover. Therefore, we selected clean latrines because that was a stand-alone question included in the survey (i.e. how many of the latrines are clean). The ‘percentage of children washing their faces when washing their hands during the school day’ is not a critical data collection point as it does not inform if the face needed to be washed. Children not washing their face during this observed moment does not directly imply dirtier faces or an improper behavior. We would therefore not recommend this indicator but rather simply ‘percentage of children with clean faces’ at defined observation points, such as arrival at the school in the morning and before leaving school to return home.

There were lessons learned from the data collection process during the pre-survey that were implemented for the post-survey to improve data quality and assurance. This included allowing data collectors and supervisors to check the data before sending to ensure increased data quality and control. In addition, mobile data capture forms were designed to validate eligibility criteria before allowing enumerators to proceed with data collection. Based on the experience from the pre-survey, a supervisor form was developed for the post-survey to calculate household replacement rates and capture the population of the village for weightings (S7 Table).

In all three countries, F&E activities funded by the Trust and DFID began before the pre-surveys were conducted, due to funding dynamics and the time it took to get survey protocols developed, approved, and implemented. This delay in pre-survey implementation creates a limitation in that the surveys might not have detected some of the changes produced by the interventions in the months before the pre-survey was conducted.

Though the F&E programs were implemented for multiple years in each country, pre and post-surveys were conducted 16 months apart in Malawi, 13 months apart in Tanzania, and 20 months apart in Uganda. As behavior change is a long process, this is likely not enough time to measure a change in behavior. The surveys were expensive due to sample sizes across multiple regions and long household survey questionnaires that collected data on a range of topics, much of which was ultimately unused or was not processed in time to be of use to programming.

### Recommendations

Based on the experience of selecting, implementing, monitoring, and evaluating F&E activities, there are several recommendations. First, the F&E activities chosen for implementation in these three countries were based on donor funding, implementing partner presence and perceptions of what was achievable in three years. Though these factors are important for program implementation, it is recommended that behavior change theory also be used to develop programs and M&E. This will not only help tailor programming to the specific needs of each community but also aid in the consistent and clear measurement directly linked to the activities. Second, ideally any future pre-surveys should occur before F&E activities are implemented if time and resources allow; however, in areas that have on-going F&E related activities, this might not be possible. Third, programs should determine what their output targets are before implementing F&E programs and when determining methods of measuring success. This should include not only change from pre and post-survey but also pre-identification of indicator levels which are considered optimal and which the maintenance of (as opposed to statistically significant increase) would be considered a successful outcome. Fourth, in order to evaluate whether activities are effective, it is important to know if they are being implemented as intended. Routine monitoring of activity implementation is critical and can provide insights and add depth to results from program evaluations. The FEME (S1, S2 and S3 Tables) provides a document that could be used as an example on which to improve. Fifth, there should be careful consideration of possible confounders of seasonality and time of day that data is collected, with efforts made to standardize the months and time of day data is collected. Sixth, unless multiple surveys are being conducted to measure longitudinal data, at least 24 months should pass between pre- and post-surveys allowing for more time for behavioral change practices to take root. Lastly, where possible, instead of conducting a standalone F&E survey, F&E data should be collected in connection with other planned programmatic, demographic, or health surveys in order to streamline human and financial resources.

## Conclusion

The application of the FEME in Malawi, Tanzania, and Uganda, and the implementation of a pre and post-survey to measure change for select F&E indicators represents an effort to fill a gap in understanding how best to evaluate F&E activities in trachoma programs. It is clear from the lessons learned and recommendations that the FEME framework, survey indicators, and survey methodologies could use some improvement or modifications to make monitoring and evaluation of F&E activities more effective. Additionally, a more robust system for monitoring implementation of F&E activities would have aided in programs making quicker programmatic decisions and allowing a better understanding of the results of the post-surveys. Despite the limitations, the experience gained from implementing the FEME, pre and post-surveys, and the supplemental materials provided in this manuscript contribute towards the effort to progress our understanding of how best to evaluate F&E activities.

## Supporting information

S1 Text Program Achievements. (DOCX)

S1 Table Uganda FEME. (XLSX)

S2 Table Malawi FEME. (XLSX)

S3 Table Tanzania FEME. (XLS)

S4 Table Household Survey. (DOCX)

S5 Table School Survey. (DOCX)

S6 Table Student Observation. (DOCX)

S7 Table Supervisor Forms. (XLSX)
