# Supplementary material for: Evaluation of facial cleanliness and environmental improvement activities: Lessons learned from Malawi, Tanzania, and Uganda
Source: PLoS Negl Trop Dis. 2021 Nov 29;15(11):e0009962. doi: 10.1371/journal.pntd.0009962 (PMC8659352; doi:10.1371/journal.pntd.0009962)
Supplement: S1 Text — (DOCX) [file pntd.0009962.s001.docx]

**Supporting Information 1: Program Achievements in Malawi, Tanzania, and Uganda**

Uganda achieved the following:

- **15,500** handwashing facilities with soap/ash and water constructed next to latrines
- **59** schools supported to develop water, sanitation, and hygiene (WASH) club activities
- **6** school latrines built
- **15,459** household latrines built
- **95** villages triggered using an adapted community led total sanitation (CLTS) hygiene focused approach with face washing and trachoma messaging
- **5** targeted villages declared open defecation free (ODF)
- **36,959** sanitation facilities rehabilitated
- **34** WASH programs incorporated face washing and trachoma messaging into existing programs
- **6** water points repaired/upgraded
- **39** water points put in place in schools
- **53,707** hygiene promoters and community leaders trained on WASH and trachoma
- **895** schools received updated School Sanitations Guidelines that included trachoma messages
- **2,611** community meetings held (including dramas performed and videos shown)
- **4,353** radio/TV spots broadcasted
- **107** radio talk shows held

Malawi achieved the following:

- **147** schools trigged using adapted school led total sanitation (SLTS) including information on trachoma
- **600** villages triggered using adapted CLTS including information on trachoma
- **2,329** patrons and leaders of school sanitation and hygiene clubs trained on WASH and trachoma
- **746** stakeholders and partners sensitized on trachoma messaging and control
- **78** WASH programs incorporated face washing and trachoma messaging into existing programs
- **3,051** health workers received training on trachoma and WASH related topics

Tanzania achieved the following:

- **80** villages triggered using an adapted CLTS hygiene focused approach with face washing and trachoma messaging
- **8** schools triggered with SLTS
- **8** schools included WASH in their school curricula
- **695** health officers at various levels trained on sanitation and hygiene (CLTS-H)
- Improved participation in WASH coordination at national and district levels by bringing in additional stakeholders
- **3,302** handwashing facilities with soap/ash and water constructed near sanitation facilities in households and schools
- **53** community sensitization campaigns/meetings held
- **10,344** sets of information, education, and communication (IEC) materials procured, designed, and developed
- **11** school health clubs established
- **112** teachers trained on National WASH Guidelines and mainstreaming of WASH issues in school curricula
