# Supplementary material for: Evaluation of facial cleanliness and environmental improvement activities: Lessons learned from Malawi, Tanzania, and Uganda
Source: PLoS Negl Trop Dis. 2021 Nov 29;15(11):e0009962. doi: 10.1371/journal.pntd.0009962 (PMC8659352; doi:10.1371/journal.pntd.0009962)
Supplement: S4 Table — (DOCX) [file pntd.0009962.s005.docx]

# Supporting Information 5: Household Survey

INTERVIEW DATE: - -

INTERVIEWER NAME: ____________________

REGION NAME: _________________________

DISTRICT NAME: ________________________

VILLAGE NAME: _________________________

CONSENT ID:____________________________

START TIME: END TIME:

**HOUSEHOLD LISTING**

I would like to have some information about the people who live in your household. Remember, by household I mean all the people slept in the house the night before. Could you give me the first initials, ages and sex of all the members of your household? Please start with yourself then the youngest, including babies. (RECORD ANSWERS IN COLUMNS 101-107 USING THE SPECIFIED CODES.)

|  | | 101 | 102 | 103 | 104 | | 105 | | 106 | | 107 | |
| --- | --- | --- | --- | --- | --- | --- | --- | --- | --- | --- | --- | --- |
|  |  | **First initial** | **Age in years** | **Sex** | **Discharge/Drainage on face^[[1]](#footnote-1)^**  ***observation*** | | **Flies on face^[[2]](#footnote-2)^**  ***observation*** | | **Dirt on face *observation*** | | **Diarrhoea^[[3]](#footnote-3)^**  **(In the last 48 hours)** | |
| **Household members (HHM)** | |  | <1 year=0  97=don’t know | Male=0  Female=1 | Y | N | Y | N | Y | N | Y | N |
| RSP | 01 |  |  |  |  |  |  |  |  |  |  |  |
| HHM | 02 |  |  |  |  |  |  |  |  |  |  |  |
| HHM | 03 |  |  |  |  |  |  |  |  |  |  |  |
| HHM | 04 |  |  |  |  |  |  |  |  |  |  |  |
| HHM | 05 |  |  |  |  |  |  |  |  |  |  |  |
| HHM | 06 |  |  |  |  |  |  |  |  |  |  |  |
| HHM | 07 |  |  |  |  |  |  |  |  |  |  |  |
| HHM | 08 |  |  |  |  |  |  |  |  |  |  |  |
| HHM | 09 |  |  |  |  |  |  |  |  |  |  |  |
| HHM | 10 |  |  |  |  |  |  |  |  |  |  |  |
| HHM | 11 |  |  |  |  |  |  |  |  |  |  |  |
| HHM | 12 |  |  |  |  |  |  |  |  |  |  |  |
| HHM | 13 |  |  |  |  |  |  |  |  |  |  |  |
| HHM | 14 |  |  |  |  |  |  |  |  |  |  |  |
| HHM | 15 |  |  |  |  |  |  |  |  |  |  |  |

| **NO** | **QUESTIONS** | | **RESPONSE OPTIONS** | | | | **SKIP TO** |
| --- | --- | --- | --- | --- | --- | --- | --- |
| **DEMOGRAPHIC INFORMATION** | | | | | | | |
|  | What is your religion? | | None…………………………………………0  Christian 1  Islam/Muslim 2  Atheist 3    Other 96  ___________________________  (SPECIFY Other)  Refused to Answer 98 | | | |  |
|  | What is your main occupation? | | Homemaker/Housewife 1  Farming 2  Fishing 3  Labourer/ Casual Worker 4  Domestic/Maid/Char/House help 5  Trader/Hawker/Vendor/Boda boda 6  Professional 7  Armed Services 8  Artisan/Skilled worker 9  Pastoralist…………………………………..10  Unemployed 11  Other .......................................................96  ___________________________  (SPECIFY Other)  Refused to Answer...................................98 | | | |  |
|  | What is the highest level of education of the head of this household? | | None 0  Informal education 1  Some but not all primary school 2  Completed Primary school 3  Some but not all secondary school 4  Completed Secondary school 5  Beyond Secondary school 6  Adult literacy 7  Vocational…………………………………. 8  Don’t know 97  Refused to answer .98 | | | |  |
|  | What is the highest level of education you completed?  (caregiver-respondent) | | None 0  Informal education 1  Some but not all primary school 2  Completed Primary school 3  Some but not all secondary school 4  Completed Secondary school 5  Beyond Secondary school 6  Adult literacy 7  Vocational………………………………….. 8  Same as head of house hold 9  Don’t know 97  Refused to answer .98 | | | |  |
|  | Are any livestock, herds, other farm animals, or poultry kept in this compound? | | No 1  Yes 2  Don’t know 97  Refused to answer 98 | | | | 301  301  301 |
|  | Where do you keep your livestock during the night?  (click all that apply) | | Outside the household compound 1  In the yard/compound of household 2  In the outside kitchen 3  In the children’s room 4  In the house 5  Other 96  __________________________  (SPECIFY Other)  Don’t know 97  Refused to answer 98 | | | |  |
| **WATER, SANITATION AND HYGIENE** | | | | | | | |
|  | What was your main source of drinking water during the past two days? | | Piped water into dwelling 1  Piped water to yard/plot 2  Public tap/standpipe 3  Tube well/borehole 4  Protected dug well 5  Unprotected dug well 6  Protected spring 7  Unprotected spring 8  Bottled water 9  Cart with small tank/drum 10  Tanker-truck 11  Surface water (river, dam, lake, pond, stream, canal, irrigation, channels) 12  Subsurface water (sand damn, scoop hole) 13  Rain water harvesting, collected in a covered place/tank 14  Rainwater collection, collected in an open place 15  Other 96  ___________________________  (SPECIFY Other)  Don’t know 97  Refused to answer 98 | | | |  |
|  | What was your main source of water for other purposes such as cooking and hand and face washing during the past two days? | | Piped water into dwelling 1  Piped water to yard/plot 2  Public tap/standpipe 3  Tubewell/borehole 4  Protected dug well 5  Unprotected dug well 6  Protected spring 7  Unprotected spring 8  Bottled water 9  Rainwater collection 11  Cart with small tank/drum 12  Tanker-truck 13  Surface water (river, dam, lake, pond, stream, canal, irrigation, channels) 14  Other .......................................................96  ___________________________  (SPECIFY Other)  Don’t know 97  Refused to answer 98 | | | |  |
|  | Is water available from this source all year round? (follow up to previous question) | | No 1  Yes 2  Don’t know 97  Refused to answer 98 | | | | 306  306  ~~306~~ |
|  | What period of time each year do you use an alternative water source? | | Less than 1 week 1  1-3 weeks........................................................2  1 month 3  2 months 4  3 months 5  4 months or more 6  Don’t Know 97  Refused to answer 98 | | | |  |
|  | What is your alternative water source? | | Piped water into dwelling 1  Piped water to yard/plot 2  Public tap/standpipe 3  Tubewell/borehole 4  Protected dug well 5  Unprotected dug well 6  Protected spring 7  Unprotected spring 8  Bottled water 9  Rainwater collection 11  Cart with small tank/drum 12  Tanker-truck 13  Surface water (river, dam, lake, pond, stream, canal, irrigation, channels) 14  Other .......................................................96  ___________________________  (SPECIFY Other)  Don’t know 97  Refused to answer 98 | | | |  |
|  | In the last two days, was water unavailable from this (main) source for a day or longer? | | No 1  Yes 2  Don’t know 97  Refused to answer 98 | | | |  |
|  | How long does it take to go there, get water, and come back (for cooking, hand and face washing)? | | No. of minutes __________  Water on premises 1  Don’t know 97  Refused to answer 98 | | | |  |
|  | Who fetched water for the household yesterday?  (Probe: is this person under 9? What sex?)  (CIRCLE ALL THAT APPLY) | | Adult woman 1  Adult man 2  Adolescent girl (9-17) 3  Adolescent boy (9-17) 4  Child girl (3-8) 3  Child boy (3-8) 4  Other 96  ___________________  (Specify)  Don’t know 97  Refused to answer 98 | | | |  |
|  | Does the household have a designated area for bathing?  (Ask, then verify with observation) | | No 1  Yes (not observed) 2  Yes (observed) 3  Don’t know 97  Refused to answer 98 | | | |  |
|  | Does your household have a hand and face washing station?  If yes ask to see hand/ face washing station | | No 1  Yes (not observed) 2  Yes (observed) 3  Don’t know 97  Refused to answer 98 | | | | 316  316  316  316 |
|  | OBSERVE: What type of hand/ face washing station is this? | | Tippy Tap 1  Leaky Tin 2  Jerrycan with tap 3  A sink 4  Bucket/cup/ basin/jerrycan...........................................5  Within toilet....................................................6  No permission to see 7  Other 96  ___________________  (Specify) | | | |  |
|  | OBSERVE: Is water present at the specific place for hand and face washing? | | No 1  Yes 2  No permission to see 3  Don’t know 97 | | | |  |
|  | OBSERVE: Is soap or ash present at the hand/ face washing station? | | No cleansing agent present 1  Soap present 2  Ashe present 3  Both soap and ashe present 4  No permission to see 5  Don’t know 97 | | | |  |
|  | OBSERVE: Where is the hand and face washing station located? | | Inside/within 10 paces of the toilet facility 1  Inside/within 10 paces of the kitchen/cooking place 2  Elsewhere in home or yard 3  Outside yard 4  No specific place 5  No permission to see 6  Other 96  ___________________  (Specify) | | | |  |
|  | OBSERVE: Accessibility  Is the hand and face washing station accessible to all people in the household? | | No 1  Yes 2  No permission to see 3  Don’t know 97 | | | |  |
|  | Did you wash your face yesterday? | | No 1  Yes 2  Don’t Remember 3  Don’t know 97  Refused to answer 98 | | | |  |
|  | Did you wash youngest child’s face yesterday? | | No 1  Yes 2  Child washes their own face 3  Don’t know 97  Refused to answer 98 | | | | 321  321  321  321 |
|  | How many times did you wash your child’s face yesterday? | | Once 1  Twice 2  Three 3  Four or more 4 | | | |  |
|  | At what moment(s), did you wash your child’s face?  (circle all that apply) | | After waking up/ morning ..............................1  Before going to school....................................2  Before going to sleep......................................3  Before eating food....................................4  After eating.....................................................5  Before religious activity..................................6  Other…………………………………….....96  ___________________________  (SPECIFY Other)  Don’t know 97  Refused to answer.......................................98 | | | |  |
|  | What did you use to wash your child’s face?  (circle all that apply) | | Water 1  Soap 2  Towel/ cloth 3  Bare hands 4  Other 96  ___________________  (Specify)  Don’t know 97  Refused to answer.......................................98 | | | |  |
|  | Ask to see the toilet facility.  OBSERVATION: What kind of toilet facility do members of your household usually use? | | Flush/pour flush to:  Piped sewer system 1  Septic tank 2  Pit latrine 3  Elsewhere 4  Unknown place/not sure/DK where 5  Ventilated improve pit latrine 6  Pit latrine with slab 7  Pit latrine without slab/open pit 8  Composting toilet 9  Bucket 10  Hanging toilet/hanging latrine 11  No facilities or bush or field 12  Other .......................................................96  ___________________________  (SPECIFY Other)  Don’t know 97  Refused to answer 98 | | | | **323**  **323** |
|  | OBSERVE: Do you see the following: | | Smooth Cleanable Floor | Lid on hole preventing flies in and out | Super structure providing privacy^[[4]](#footnote-4)^ | Presence of cleansing materials in latrine | **All skip to 324** |
|  |  |  | Excellent.....1  Acceptable.2  Not acceptable.3 | No.........1  Yes.......2  Unable to determine.........3 | No............1  Yes..........2  Unable to determine.3 | No...........1  Yes..........2  Unable to determine.3 |  |
|  |  |  | Clean^[[5]](#footnote-5)^ | Latrine full ^[[6]](#footnote-6)^ | Within ~10 metres from the house | accessible to all members of the household^[[7]](#footnote-7)^ |  |
|  |  |  | Excellent......1  Acceptable...2  Not acceptable…3 | No.........1  Yes.......2  Unable to determine.........3 | No............1  Yes..........2  Unable to determine.3 | No............1  Yes..........2  Unable to determine.3 |  |
|  | What is done to dispose of stools?  CIRCLE ALL THE APPLY | | Burned 1  Put/rinsed into toilet/latrine 2  Put/rinsed into drain or ditch 3  Thrown into garbage 4  Buried/ covered 5  Left in the open 6  Other .......................................................96  ___________________________  (SPECIFY Other)  Don’t know 97  Refused to answer 98 | | | | **All skip to 328** |
|  | Which household members used this facility in the past two days?  (circle all that apply) | | No one.....................................................................0  Elderly women (over 70)........................................1  Elderly men (over 70)..............................................2  Adult (non-elderly) women......................................3  Adult (non-elderly) men ..........................................4  Adolescent boys (9-15 years-old) ............................5  Adolescent girls (9-15 years-old) .............................6  Girls (3-9 years-old) .................................................7  Boys (3-9 years-old) .................................................8  Don’t know 97  Refused to answer 98 | | | |  |
|  | Do all members of this household use this toilet? | | No ……………………………………………1  Yes 2  Don’t know....................................................97 | | | | **327**  **327** |
|  | Who does not use the facility? | | Elderly women (over 70) ..........................................1  Elderly men (over 70)................................................2  Adult (non-elderly) women......................................3  Adult (non-elderly) men ..........................................4  Pregnant women.....................................................5  Adolescent boys (9-15 years-old) ............................6  Adolescent girls (9-15 years-old) .............................7  Girls (3-9 years-old) .................................................8  Boys (3-9 years-old) .................................................9  Everyone uses the facility……………………..10  Don’t know 97  Refused to answer 98 | | | |  |
|  | The last time the youngest child passed stools, what was done to dispose of the stools? | | Child used toilet/latrine 1  Put/rinsed into toilet/latrine 2  Put/rinsed into drain or ditch 3  Thrown into garbage 4  Buried 5  Left in the open 6  Other 96  ___________________________  (SPECIFY Other)  Don’t know 97  Refused to answer 98 | | | |  |
|  | Do you share this toilet with other households? | | No 1  Yes 2  Don’t know 97  Refused to answer 98 | | | |  |
|  | How do you dispose of rubbish from your household? | | Burn 1  Bury 2  Dump into a pit 3  Dump in the backyard 4  Dump in front of the house 5  Refuse heap within community 6  Refuse heap outside of community 7  Other 96  _________________________  (SPECIFY Other)  Don’t know 97  Refused to answer 98 | | | |  |
|  | OBSERVE: Overall cleanliness of compound | | Human feces in compound | | Trash/rubbish throughout compound | |  |
|  |  |  | No................................1  Yes...............................2  Unable to determine...3 | | No................................1  Yes...............................2  Unable to determine...3 | |  |
|  |  |  | Refuse heap within compound for discarding rubbish | | Compound is well swept | |  |
|  |  |  | No................................1  Yes...............................2  Unable to determine...3 | | No................................1  Yes...............................2  Unable to determine...3 | |  |
| **HYGIENE KNOWLEDGE** | | | | | | | |
| 401 | | Please mention all of the occasions when it is important to wash (clean) your face?  (CIRCLE ALL THAT APPLY) | When you wake up 1  Before you go to sleep 2  After eating 3  Face is visibly dirty 4  Nose running 5  Runny eyes 6  When leaving the compound 7  Before prayer 8  Other 96  ___________________  (Specify)  Don’t know 97  Refused to answer 98 | | | |  |
|  | | Please mention all of the occasions when it is important to wash (clean) your children’s faces?  (CIRCLE ALL THAT APPLY) | When they wake up 1  Before they go to sleep 2  After eating 3  Face is visibly dirty 4  Nose running 5  Runny eyes 6  When leaving the compound 7  Before going to school 8  Other 96  ___________________  (Specify)  Don’t know 97  Refused to answer 98 | | | |  |
|  | | Please mention all of the occasions when is it important to wash your hands with soap?  (CIRCLE ALL THAT APPLY) | Before eating 1  After eating 2  Before praying 3  Before breastfeeding or feeding a child 4  Before cooking or preparing food 5  After defecation/urination 6  After cleaning a child that has defecated/changing a child’s nappy 7  When my hands are dirty 8  After cleaning the toilet or potty 9  Other 10  ___________________  (Specify)  Don’t know 97  Refused to answer 98 | | | |  |
| **KNOWLEDGE, RISK, and EFFICACY** | | | | | | | |
|  | Have you heard of trachoma/local term for trachoma? | | No 1  Yes 2  Refused to answer 98 | | | | **517** |
|  | What part of the body does it affect? | | Eyes 1  Chest 2  Stomach 3  Limbs 4  Genitals 5  Other 96  ___________________________  (SPECIFY Other)  Don’t know 97  Refused to answer 98 | | | |  |
|  | How does a person get trachoma?  (CIRCLE ALL THAT APPLY) | | A disease caused by flies 1  Coming in contact with an infected person  2  Using a dirty cloth of someone infected with trachoma 3  Runs in the family 4  Evil spirits 5  Other 96  ___________________  (Specify)  Don’t know 97  Refused to answer 98 | | | |  |
|  | What are the signs and symptoms of trachoma?  (CIRCLE ALL THAT APPLY) | | Runny eyes 1  Itchy eyes 2  Turned in eyelashes 3  There are none 4  Blindness……………………………………5  Other 96  ___________________  (Specify)  Don’t know 97  Refused to answer 98 | | | |  |
|  | Have you or anyone in your household suffered from trachoma/local word? | | No 1  Yes 2  Don’t know 97  Refused to answer 98 | | | |  |
|  | What can you do to treat trachoma?  (CIRCLE ALL THAT APPLY) | | Surgery 1  Epilation..........................................................2  Take medicine 3  Herbal medicines/salves 4  There is no treatment 5  Other 96  ___________________  (Specify)  Don’t know 97  Refused to answer 98 | | | |  |
|  | Are you currently doing anything to prevent you and your family from getting trachoma? | | No 1  Yes 2  Don’t know 97  Refused to answer 98 | | | | **509**  **509**  **509** |
|  | What are you doing to prevent you and your family from getting trachoma?  (CIRCLE ALL THAT APPLY) | | Washing your face 1  Washing your child’s face 2  Washing your hands 3  Washing your child’s hands 4  Taking medicine during a MDA 5  Fly control measures 6  Using a latrine 7  Keeping compound clean 8  Taking medicine from clinic 9  Other 96  ___________________  (Specify)  Don’t know 97  Refused to answer 98 | | | |  |
|  | Do you feel that people who live in this community are at risk for getting trachoma? | | No 1  Yes 2  Don’t know 97  Refused to answer 98 | | | |  |
|  | What groups of people are most at risk for getting trachoma?  (CIRCLE ALL THAT APPLY) | | Elderly 1  Children 2  Women 3  Mother/Caretakers 4  Other 96  ___________________  (Specify)  Don’t know 97  Refused to answer 98 | | | |  |
|  | Are you and your family at risk for getting trachoma? | | No 1  Yes 2  Don’t know 97  Refused to answer 98 | | | |  |
|  | What puts someone at risk for getting trachoma?  (CIRCLE ALL THAT APPLY) | | Lack of water 1  Lack of soap 2  Dirty face or hands 3  Poverty 4  No functioning toilet 5  Crowded household 6  Low education 7  Nature of Employment 8  Dirty environment 9  Other 96  ___________________  (Specify)  Don’t know 97  Refused to answer 98 | | | |  |
|  | What helps to reduce the risk of you getting trachoma?  (CIRCLE ALL THAT APPLY) | | Washing your face 1  Washing your child’s face 2  Washing your hands 3  Washing your child’s hands 4  Participating in MDA 5  Fly control measures 6  Clean environment/ compound 7  Other 96  ___________________  (Specify)  Don’t know 97  Refused to answer 98 | | | |  |
|  | Out of 10 people you know in your community, how many people do you think will get trachoma? | | __________________  (Number)  Don’t know 97  Refused to answer 98 | | | |  |
|  | How confident are you that you can protect yourself and your family from becoming infected with trachoma?  (Read options out loud) | | Extremely confident 1  Somewhat confident 2  Not very confident 3  Not confident at all 4  Don’t know 97  Refused to answer 98 | | | |  |
|  | How confident are you that your community can prevent trachoma?  (Read options out loud) | | Extremely confident 1  Somewhat confident 2  Not very confident 3  Not confident at all 4  Don’t know 97  Refused to answer 98 | | | |  |
|  | How confident are you that you can practice healthy hygiene habits?  (Read options out loud) | | Extremely confident 1  Somewhat confident 2  Not very confident 3  Not confident at all 4  Don’t know 97  Refused to answer 98 | | | |  |
| **MEDIA/COMMUNICATION** | | | | | | | |
|  | How often do you read a newspaper or magazine?  Read out answer choices | | Almost every day 01  At least once a week 02  Not often 03  Not at all 04  Don’t know 97  Refused to answer 98 | | | |  |
|  | How often do you listen to the radio?  Read out answer choices | | Almost every day 01  At least once a week 02  Not often 03  Not at all 04  Don’t know 97  Refused to answer 98 | | | |  |
|  | How often do you watch television?  Read out answers | | Almost every day 01  At least once a week 02  Not often 03  Not at all 04  Don’t know 97  Refused to answer 98 | | | |  |
|  | How do people in this community usually receive health information?  (CIRCLE ALL THAT APPLY) | | Community meetings 1  From the church/ mosques 2  From other community members 3  From village health team (VHT) 4  From School teachers....................................5  Traditional healer 6  Newspapers, magazines, brochures 7  Radio 8  Television 9  Roaming car with megaphone 10  Health facility/ worker 11  Community programs 12  Other 96  ___________________  (Specify)  Don’t know 97  Refused to answer 98 | | | |  |
|  | What are the top three (3) health concerns in your community?  (CIRCLE THREE) | | Common cold 1  Malaria 2  Diarrhea 3  Cholera 4  HIV 5  Tuberculosis 6  Trachoma 7  Pneumonia 8  Jiggers 9  No other diseases 10  Other 96  ___________________  (Specify)  Don’t know 97  Refused to answer 98 | | | |  |
|  | What information would you like to receive about trachoma?  (CIRCLE ALL THAT APPLY) | | Information about how it is spread 1  Signs and symptoms 2  How to prevent trachoma 3  How to treat trachoma 4  Where to go to get treated 5  Other 96  ___________________  (Specify)  Don’t know 97  Refused to answer 98 | | | |  |
| **MESSAGES AND RECALL** | | | | | | | |
|  | In the past 2 weeks, who have you talked about trachoma with?  (CIRCLE ALL THAT APPLY) | | Husband/wife 1  Mother-in-law/father-in-law 2  Parents 3  Brother or sister 4  Children 5  Other family member, relative 6  Friend(s) 7  Health worker 8  Children 9  No one 10  Community leader 11  Other 96  ___________________  (Specify)  Don’t know 97  Refused to answer 98 | | | | **703**  **703**  **703** |
|  | What did you talk about ?  (CIRCLE ALL THAT APPLY) | | Surgery 1  MDA 2  Signs of trachoma 3  Symptoms of trachoma 4  Someone who had trachoma 5  Ways to prevent trachoma 6  Face washing 7  Environmental cleanliness 8  Using a latrine 9  Other 96  ___________________  (Specify)  Don’t know 97  Refused to answer 98 | | | |  |
|  | In the last two weeks have you seen, heard, or watched any program or read any document about trachoma? | | No 1  Yes 2  Don’t know 97  Refused to answer 98 | | | | **END**  **END**  **END** |
|  | What was the source of this information? (CIRCLE ALL THAT APPLY) | | Radio 1  Television/video 2  Mobile phone 3  Newspaper or magazine 4  Posters/billboards 5  Community drama 6  Community event/meeting 7  Community health worker 8  Roaming car with megaphone 9  Children 10  Other 96  ___________________  (Specify)  Don’t know 97  Refused to answer 98 | | | | **END** |
|  | Did you do anything different after listening to the message? | | No 1  Yes 2  Don’t know 97  Refused to answer 98 | | | | **END**  **END**  **END** |
|  | What did you do?  (CIRCLE ALL THAT APPLY) | | Sought treatment at clinic 1  Consulted health professional 2  Discussed with family or friends 3  Other 96  ___________________  (Specify)  Don’t know 97  Refused to answer 98 | | | |  |

Thank the respondent for their time. Respond to any questions that they may.

1. Look for any mucous and ocular or nasal discharge on the face [↑](#footnote-ref-1)
2. Look for any flies that are landing on the face at time of observation [↑](#footnote-ref-2)
3. Diarrheoa means three or more loose stools per day [↑](#footnote-ref-3)
4. At least three walls (4 ft. high) and door [↑](#footnote-ref-4)
5. Clean means there is no excessive smell, there are no visible feces in or around the facility, there are no flies and there is no litter. [↑](#footnote-ref-5)
6. Assumed if unable to observe and not reported [↑](#footnote-ref-6)
7. Facility is accessible to all members of the household with a focus on children, elderly, and people with disabilities [↑](#footnote-ref-7)
