# Supplementary material for: Evaluation of facial cleanliness and environmental improvement activities: Lessons learned from Malawi, Tanzania, and Uganda
Source: PLoS Negl Trop Dis. 2021 Nov 29;15(11):e0009962. doi: 10.1371/journal.pntd.0009962 (PMC8659352; doi:10.1371/journal.pntd.0009962)
Supplement: S5 Table — (DOCX) [file pntd.0009962.s006.docx]

**Supporting Information 6: School Survey**

INTERVIEW DATE: - -

INTERVIEWER NAME: ____________________

REGION NAME: ________________________

DISTRICT NAME: ______________________

SCHOOL NAME: ________________________

CONSENT ID:___________________________

SCHOOL TYPE : Day □ Boarding □ Both

Gender of pupils: Mixed □ Boys □ Girls □

Function of respondent (headmaster, head teacher, etc.). ____________________________

| **DEMOGRAPHICS** | | | | | | | | | | | | | | | |
| --- | --- | --- | --- | --- | --- | --- | --- | --- | --- | --- | --- | --- | --- | --- | --- |
|  | | **P1** | **P2** | | **P3** | **P4** | | **P5** | | | **P6** | **P7** | | **P8** | |
| 101. Total boys enrolled | |  |  | |  |  | |  | | |  |  | |  | |
| 102. Total girls enrolled | |  |  | |  |  | |  | | |  |  | |  | |
| 103. Total boys present today | |  |  | |  |  | |  | | |  |  | |  | |
| 104. Total girls present today | |  |  | |  |  | |  | | |  |  | |  | |
| 105.Total male teachers | |  |  | |  |  | |  | | |  |  | |  | |
| 106. Total female teachers | |  |  | |  |  | |  | | |  |  | |  | |
| **WATER, SANITATION AND HYGIENE** | | | | | | | | | | | | | | | |
|  |  | | |  | | | | | | | | | | | **Skip to** |
| 201. | What is the school’s main water source? | | | Piped water into school building 1  Piped water to school yard/plot 2  Public tap/standpipe 3  Tubewell/borehole 4  Protected dug well 5  Unprotected dug well 6  Protected spring 7  Unprotected spring 8  Bottled water 9  Rainwater collection 10  Cart with small tank/drum 11  Tanker-truck 12  Surface water (river, dam, lake, pond, stream, canal, irrigation, channels) 13  No water available in or near school 14  Other 96  ___________________________  (SPECIFY Other)  Don’t know 97  Refused to answer 98 | | | | | | | | | | |  |
| 202. | While school is in session, is water always available from this source? | | | No 1  Yes 2  Don’t know 97  Refused to answer 98 | | | | | | | | | | | **204** |
| 203. | If there is no water available in or near school, where do you get water from? | | | Students bring water to school 1  Teacher brings water to school 2  Other 96  ___________________________  (SPECIFY Other)  Don’t know 97  Refused to answer 98 | | | | | | | | | | |  |
| 204. | How many days per week is the water source functional? | | | 5-7 days per week 1  2-4 days per week 2  Fewer than 2 days per week 3  Don’t know 97  Refused to answer 98 | | | | | | | | | | |  |
| 205. | How do you dispose of waste from your school? | | | Burn 1  Bury 2  Dump in the gullies 3  Dump in the backyard 4  Dump in front of the school 5  Refuse heap within school 6  Refuse heap outside of school 7  Other 96  _________________________  (SPECIFY Other)  Don’t know 97  Refused to answer 98 | | | | | | | | | | |  |
| 206. | Does the school have any latrines? | | | No 1  Yes 2  Don’t know 97  Refused to answer 98 | | | | | | | | | | | **211**  **211**  **211** |
| 207. | How many toilets are there in the school for children. | | | Exclusively for girls | | | | | (number) | | | | | |  |
|  |  |  |  | Exclusively for boys | | | | | (number) | | | | | |  |
|  |  |  |  | Unisex | | | | | (number) | | | | | |  |
| 208. | ASK TO SEE THE LATRINES  (OBSERVE)  FOR GIRLS: Observe the latrines and record how many latrines have the following: | | | Lid on hole preventing flies in and out  _________  (number) | | | Visible Feces  _________  (number) | | | Clean^[[1]](#footnote-1)^  ________  (number) | | | Latrine full ^^[[2]](#footnote-2)^^  _________  (number) | |  |
|  |  |  |  | Accessible to all children at school^^[[3]](#footnote-3)^^  _________  (number) | | | Cleansing Material available  No................................1  Yes...............................2  Unable to determine...3 | | | | | |  | |  |
| 209 | (OBSERVE)  FOR BOYS: Observe the latrines and record how many latrines have the following: | | | Lid on hole preventing flies in and out  _________  (number) | | | Visible Feces  _________  (number) | | | Clean^[[4]](#footnote-4)^  ________  (number) | | | Latrine full ^^[[5]](#footnote-5)^^  _________  (number) | |  |
|  |  |  |  | Accessible to all children at school^^[[6]](#footnote-6)^^  _________  (number) | | | Cleansing Material available  No................................1  Yes...............................2  Unable to determine...3 | | | | | |  | |  |
| 210. | (OBSERVE)  UNISEX: Observe the latrines and record how many latrines have the following: | | | Lid on hole preventing flies in and out  _________  (number) | | | Visible Feces  _________  (number) | | | Clean^[[7]](#footnote-7)^  ________  (number) | | | Latrine full ^^[[8]](#footnote-8)^^  _________  (number) | |  |
|  |  |  |  | Accessible to all children at school^^[[9]](#footnote-9)^^  _________  (number) | | | Cleansing Material available  No................................1  Yes...............................2  Unable to determine...3 | | | | | |  | |  |
| 211. | Observe overall cleanliness of school compound | | | Visible human feces in compound | | | | | | Trash/rubbish throughout compound | | | | |  |
|  |  |  |  | No................................1  Yes...............................2  Unable to determine...3 | | | | | | No................................1  Yes...............................2  Unable to determine...3 | | | | |  |
|  |  |  |  | Refuse heap within compound for discarding waste | | | | | | Compound is well swept | | | | |  |
|  |  |  |  | No................................1  Yes...............................2  Unable to determine...3 | | | | | | No................................1  Yes...............................2  Unable to determine...3 | | | | |  |
| 212. | Do teachers have their own latrines (separate from children’s facilities)? | | | No 1  Yes 2  Don’t know 97  Refused to answer 98 | | | | | | | | | | |  |
| 213. | Does the school have hand and face washing facilities? (check one) | | | No 1  Yes 2  Don’t know 97  Refused to answer 98 | | | | | | | | | | | **218**  **218**  **218** |
| 214. | How many hand and face washing stations are there in the school: (insert number) | | | Group hand/face washing station | | | | | | Individual hand/face/washing station | | | | |  |
|  |  |  |  | (number) | | | | | | (number) | | | | |  |
| 215. | (OBSERVE)  Out of all the hand and face washing stations, how many have soap available? | | | (number) | | | | | | | | | | |  |
| 216. | (OBSERVE)  Are hand and face washing stations accessible to children with physical disabilities? (check one) | | | None are 1  Some 2  Yes, all facilities are accessible 3  Don’t know 97  Refused to answer 98 | | | | | | | | | | |  |
| 217. | (OBSERVE)  Where are the hand and face washing stations located?  (SELECT ALL THAT APPLY) | | | Inside/within 10 paces of the toilet facility  1  Near the entrance of the school 2  Near the entrance to Classroom 3  No specific place 4  Other 96  ___________________  (Specify) | | | | | | | | | | |  |
| 218. | Is there a school health club that meets regularly at your school? | | | No 1  Yes 2  YES (But does not meet regularly)^[[10]](#footnote-10)^................3  Don’t know 97  Refused to answer 98 | | | | | | | | | | |  |
| 219. | Is hygiene taught in this school? | | | No 1  Yes 2  Don’t know 97  Refused to answer 98 | | | | | | | | | | | **222**  **222**  **222** |
| 220. | Who is responsible for teaching hygiene? | | | Health club 1  Main Classroom teacher 2  Dedicated health or science teacher 3  Environmental health officer 4  Village health worker 5  Other 96  ___________________________  (SPECIFY Other)  Don’t know 97  Refused to answer 98 | | | | | | | | | | |  |
| 221. | What is taught?  (CIRCLE ALL THAT APPLY) | | | Hand washing 1  Face washing 2  Overall body hygiene 3  Dental hygiene 4  Importance of sanitation 5  Other 96  ___________________________  (SPECIFY Other)  Don’t know 97  Refused to answer 98 | | | | | | | | | | |  |
| 222. | Is trachoma messaging integrated into WASH lessons? | | | No 1  Yes 2  Don’t know 97  Refused to answer 98 | | | | | | | | | | | **END**  **END**  **END** |
| 223. | If yes what are the messages? | | | Wash hands  Wash face  General hygiene  Fly control measures  Clean environment/compound  Other 96  ___________________________  (SPECIFY Other)  Don’t know 97  Refused to answer 9 | | | | | | | | | | |  |

1. Clean means there is no excessive smell, there are no visible feces in or around the facility, there are no flies and there is no litter. [↑](#footnote-ref-1)
2. Assumed if unable to observe and not reported [↑](#footnote-ref-2)
3. Facility is accessible to students with a focus on people with disabilities [↑](#footnote-ref-3)
4. Clean means there is no excessive smell, there are no visible feces in or around the facility, there are no flies and there is no litter. [↑](#footnote-ref-4)
5. Assumed if unable to observe and not reported [↑](#footnote-ref-5)
6. Facility is accessible to students with a focus on people with disabilities [↑](#footnote-ref-6)
7. Clean means there is no excessive smell, there are no visible feces in or around the facility, there are no flies and there is no litter. [↑](#footnote-ref-7)
8. Assumed if unable to observe and not reported [↑](#footnote-ref-8)
9. Facility is accessible to students with a focus on people with disabilities [↑](#footnote-ref-9)
10. Regularly meets at least once per month [↑](#footnote-ref-10)
