# Supplementary material for: Evaluation of facial cleanliness and environmental improvement activities: Lessons learned from Malawi, Tanzania, and Uganda
Source: PLoS Negl Trop Dis. 2021 Nov 29;15(11):e0009962. doi: 10.1371/journal.pntd.0009962 (PMC8659352; doi:10.1371/journal.pntd.0009962)
Supplement: S6 Table — (DOCX) [file pntd.0009962.s007.docx]

**Supporting Information 7: Student Observation**

**Aim:** To determine face-washing practices

**Preparatory materials required:**

- Observation sheets
- Pencil and rubber
- School codes

**Specific instructions for tool:**

- The Evaluation Assistant is expected to spend a minimum of three hours at each of the schools. More than one observation sheet can be used per school but you must keep track of the number of sheets used and record the final number in the top right hand corner. You must also note the school code on each separate sheet in case they become loose, as this will make it easier to ensure there is no loss of data.
- A separate line should be filled for each separate handwashing and face-washing event that occurred. For the purposes of this evaluation, if a child washes their face **at the same time** as they wash their hands, this is one event and should be captured on one line. If they wash their hands only, this is also one event and should be captured on a separate line. If they wash their face only, this is also one event and should be captured on a separate line. We want to be able to capture if the children are washing their faces at the same time as they are washing their hands.
